# A meta‐analysis of comparisons of various surgical treatments for moyamoya diseases

**DOI:** 10.1002/brb3.2356

**Published:** 2021-09-14

**Authors:** Kai Lin, Shaohua Sui, Jing Zhao, Liyong Zhang, Kun Chen

**Affiliations:** ^1^ Department of Neurosurgery Liaocheng People's Hospital Liaocheng P.R. China; ^2^ Department of Emergency Liaocheng People's Hospital Liaocheng P.R. China; ^3^ Department of Pediatrics Liaocheng People's Hospital Liaocheng P.R. China; ^4^ Department of Neurology Liaocheng People's Hospital Liaocheng P.R. China

**Keywords:** combined bypass, direct bypass, indirect bypass, moyamoya disease

## Abstract

**Purpose:**

Ischemia is one of the most familiar complications in the different procedures for moyamoya disease (MMD), but the optimal surgical approaches for MMD remain unknown. We aimed to evaluate the efficiency of various surgical treatments.

**Methods:**

A literature search word was performed through four databases such as Cochrane Library, Web of Science, PubMed, and EMBASE for the literature published until May 2021. The *I* ^2^ statistic was used to assess heterogeneity. A random/fixed‐effects model was used to pool.

**Results:**

There are a total of 18 studies including three surgical treatments such as including indirect, direct, and combined bypass in this study. The result revealed that indirect bypass was related to a higher incidence of recurrence stroke compared to the direct and combined bypass treatment (*p* = .001). Furthermore, the cases undergoing direct bypass were associated with a better angiographic change than the indirect bypass (OR = 3.254, *p* = .013).

**Conclusion:**

This meta‐analysis demonstrated a positive effect of using the direct and combined bypass to treat MMD compared to indirect bypass due to their lower rates of recurrence stroke.

## 1 INTRODUCTION

Moyamoya disease (MMD) was first reported by Japanese literature in 1957 (Suzuki & Takaku, 1969), which was characterized by progressive stenosis and eventual occlusion of the major intracranial arteries in the proximity of the distal ends of internal carotid arteries (ICA). As a result of progressive blockage of the major vessels, networks of small collateral vessels develop and show a “puff of smoke” appearance on angiography, for which the name “moyamoya” (Japanese word for “puff of smoke”) was coined (Suzuki & Takaku, 1969). MMD can cause strokes including two main phenotypes in populations: the ischemic and hemorrhagic types. Severe neuropsychological disorders, including cognitive decline, depression, and anxiety that cause the quality of life decline are also the main clinical feature of MMD (Cho et al., 2015). It is reported that MMD is common in East Asian people such as Korean and Japanese, as compared to Western Hemisphere people. According to a survey performed in Japan, the prevalence of MMD was approximately 3.16/100,000 (Wakai et al., 1997). The Japanese literature has reported that the prevalence of MMD had doubled from 3900 in 1994 to 7700 in 2003 (Kainth et al., 2013). The serious complications of MMD can lead to worse clinical outcomes and an increased mortality rate in MMD patients. Despite the absence of supporting evidence from large randomized prospective clinical trials, there is growing scientific evidence and acceptance that surgery revascularization is the most effective treatment for patients with MMD (Guzman et al., 2009; Kuroda & Houkin, 2008). Different revascularization strategies are available which can conceptionally be divided into three main categories, namely direct bypass (DB), indirect bypass (IB), and combined bypass (CB) (Kuroda & Houkin, 2008). To date, this has not been studied systematically whether direct, indirect, or combined procedures of revascularization will result in a best extensive collateral blood supply, and therefore provide better protection for the ischemic brain. Therefore, this systematic review and meta‐analysis aimed to summarize and critically appraise all existing evidence on the clinical outcome for the treatment of MMD in a large series of patients who underwent various types of surgical procedures.

## 2 METHODS

This study was based on the acknowledged PRISMA guidelines (the prioritized reported items for systematic review and meta‐analysis).

## 3 ETHICAL REVIEW

All analyses were conducted according to the available published literature; thus, no ethical approval or patient consent was required.

### 3.1 Literature and search strategy

The electronic databases, including Cochrane Library, Web of Science, PubMed, and EMBASE were retrieved to identify the study exploring effects and safety of different surgical revascularization of MMD in the clinical course of MMD patients from the inception of electronic databases to May 2021. Structured search strategies were used in combination as shown in Table 1, according to Boolean logic. In addition, the research on the appraisal reference list was manually reviewed for other potential trials that should be included. The process was iterated until no further articles could be determined.

**TABLE 1:** The search strategy

Surgical treatment OR direct bypass surgery OR indirect bypass surgery OR combined surgery OR STA‐MCA anastomosis OR multiple burr holes OR encephaloduroarteriosynangiosis (EDAS) OR omental transplantation OR superficial temporal artery to middle cerebral artery (STA‐MCA) anastomosis bypass with EDAMS OR encephaloduroarteriomyosynangiosis SMA with encephalomyosynangiosis (EMS)	
AND	
Moyamoya disease OR MMD	
AND	
Recurrent ischemic symptoms OR recurrent hemorrhage OR recurrence stroke OR postoperative complication OR postoperative death OR good angiographic change OR modified Rankin Scale	

### 3.2 Inclusion and exclusion criteria

If the article met the following criteria following PICOS, the article was considered to be included in the current meta‐analysis: (I) patients with MMD; (II) surgical revascularizations; (III) DB, IB, and CB surgery; (IV) one or more of the preplanned outcomes were reported; (V) an official published full‐text English‐written article. Case reports, animal studies, comments, letters, editorials, protocols, guidelines, and review papers were excluded.

### 3.3 Data extraction

Two of the authors independently extracted data from all the included studies. The following essential information was captured: the first author's name, publication year, sample size, study design, and outcomes. Other relevant data such as patient characteristics and literature quality scores were also extracted from individual studies.

### 3.4 Data synthesis and analysis

All meta‐analyses of eligible results were conducted using the STATA version 12.0 (Stata Corporation, College Station, Texas, USA). Heterogeneity among studies was estimated using a χ^2^ test, and the *I* ^2^ value was identified to describe the percentage variance in trials attributable to heterogeneity. *I* ^2^ > 50% was deemed as the high heterogeneity, and a random‐effect model was applied. Otherwise, the fixed‐effect model was chosen. The odds ratios (ORs) or rate differences (RDs) with 95% confidence intervals (CIs) were applied for the evaluation of binary variables, and *p*‐value < 0.05 was regarded as statistically significant.

## 4 RESULTS

### 4.1 Search results

**FIGURE 1:**
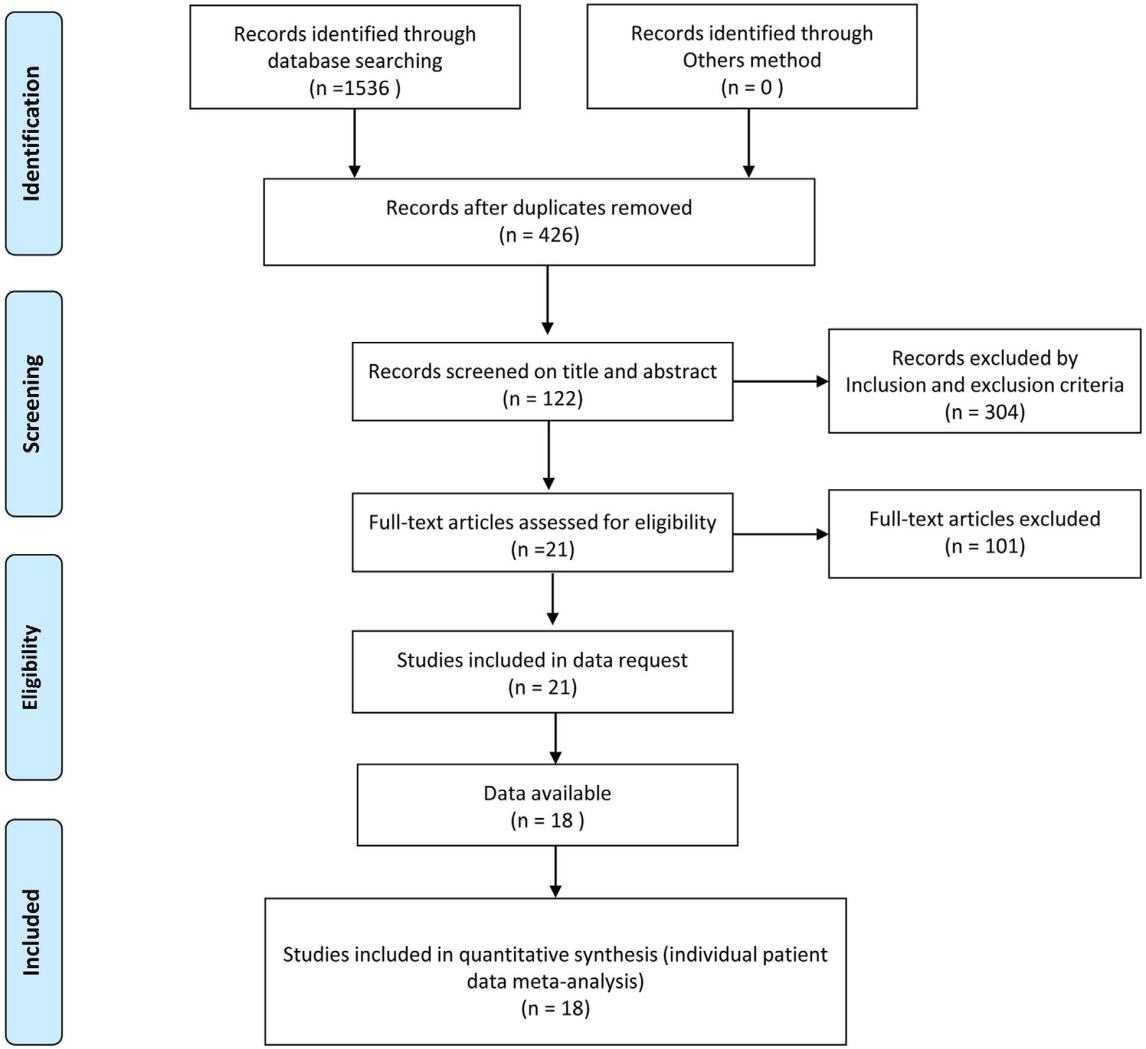
. Flowchart of the study selection process

The selection process is illustrated in Figure 1, and 1536 articles were searched in the original databases. Of these records, 1110 publications were removed owing to duplication. Meanwhile, 304 publications were eliminated due to different reasons. Full‐text of the remaining 122 publications were assessed for eligibility. Three articles were excluded since they did not compare one or more of the preplanned outcomes. Finally, there were 18 articles involved in our quantitative synthesis (Bang et al., 2012; Bot et al., 2018; Choi et al., 2013; Deng et al., 2018; Golby et al., 1999; Huang et al., 2015; Kawaguchi et al., 2000; Kim et al., 2012; Lee et al., 2012; X. Liu et al., 2013; Matsushima et al., 1998, 1992; Mesiwala et al., 2008; Zhai et al., 2018; M. Zhao et al., 2017; Y. Zhao et al., 2019, 2018; Zheng et al., 2019).

### 4.2 Quality assessment

The Newcastle‐Ottawa Scale (NOS) was taken to evaluate the quality of included studies. Two authors have appraised the quality of all involved studies. The differences that arose in the process were solved by discussing. More details about the specific scores are shown in Table 2.

**TABLE 2:** Summary of study characteristics

				Participants (n)			Gender (F/M)			NOS
Author	Country	Years	Type of study	DB	IB	CB	DB	IB	CB	Scores
Liu et al.	China	2013	Retrospective	29	12	11	NA	NA	NA	6
Y. Zhao et al.	China	2019	Retrospective	34	104	NA	16/18	51/53	NA	7
Huang et al.	China	2015	Retrospective	59	34	31	38/21	25/9	19/12	7
Bang et al.	Korea	2011	Retrospective	11	13	47	NA	NA	NA	7
Choi et al.	Korea	2013	Retrospective	9	18	8	5/4	11/7	4/4	7
Kawaguchi et al.	Japan	2000	Retrospective	6	5	NA	5/1	3/2	NA	7
Lee et al.	Korea	2012	Retrospective	27	68	29	14/13	43/25	20/9	7
Mesiwala et al.	USA	2008	Retrospective	36	3	NA	NA	NA	NA	8
Matsushima et al.	Japan	1992	Retrospective	7	13	NA	NA	NA	NA	8
Deng et al.	China	2017	Prospective	241	207	81	116/125	124/83	41/40	8
M. Zhao et al.	China	2017	Retrospective	NA	53	42	NA	27/26	20/22	7
Zhai et al.	China	2018	Retrospective	NA	21	117	NA	NA	NA	8
Kim et al.	Korea	2012	Retrospective	NA	45	51	NA	32/13	36/15	8
Matsushima et al.	Japan	1998	Prospective	16	12	22	NA	NA	NA	7
Alexandra et al.	USA	1999	Retrospective	12	NA	4	5/7	NA	1/3	8
Zheng et al.	China	2019	Retrospective	47	150	17	26/21	75/75	6/11	7
Y. Zhao et al.	China	2018	Retrospective	17	NA	54	13/4	NA	27/27	8
Bot et al.	USA	2019	Retrospective	NA	1	10	0	1/0	7/3	7

Abbreviations: CB, combined bypass surgery; DB, direct bypass surgery; IB, indirect bypass surgery; NA, not available; NOS, Newcastle‐Ottawa Scale.

### 4.3 Study characteristics

Demographic characteristics concerning the included studies are summarized in Table 2. The studies are from China, Korea, Japan, and the USA from 1992 to 2019 and involved 1834 patients (551 in DB, 759 in IB, and 524 in CB group). Four studies are showing the comparisons between DB and IB surgery and involved 208 patients (83 in DB and 125 in the IB group). Four publications compare the effects between the IB and CB group including 340 patients (120 are from IB, 220 in CB). Eight pieces of literature conducted the comparison about all three bypass surgeries and involved 1199 patients (439 from DB, 514 from IB, and 246 from the CB group).

### 4.4 Outcomes

#### 4.4.1 DB versus IB

**FIGURE 2:**
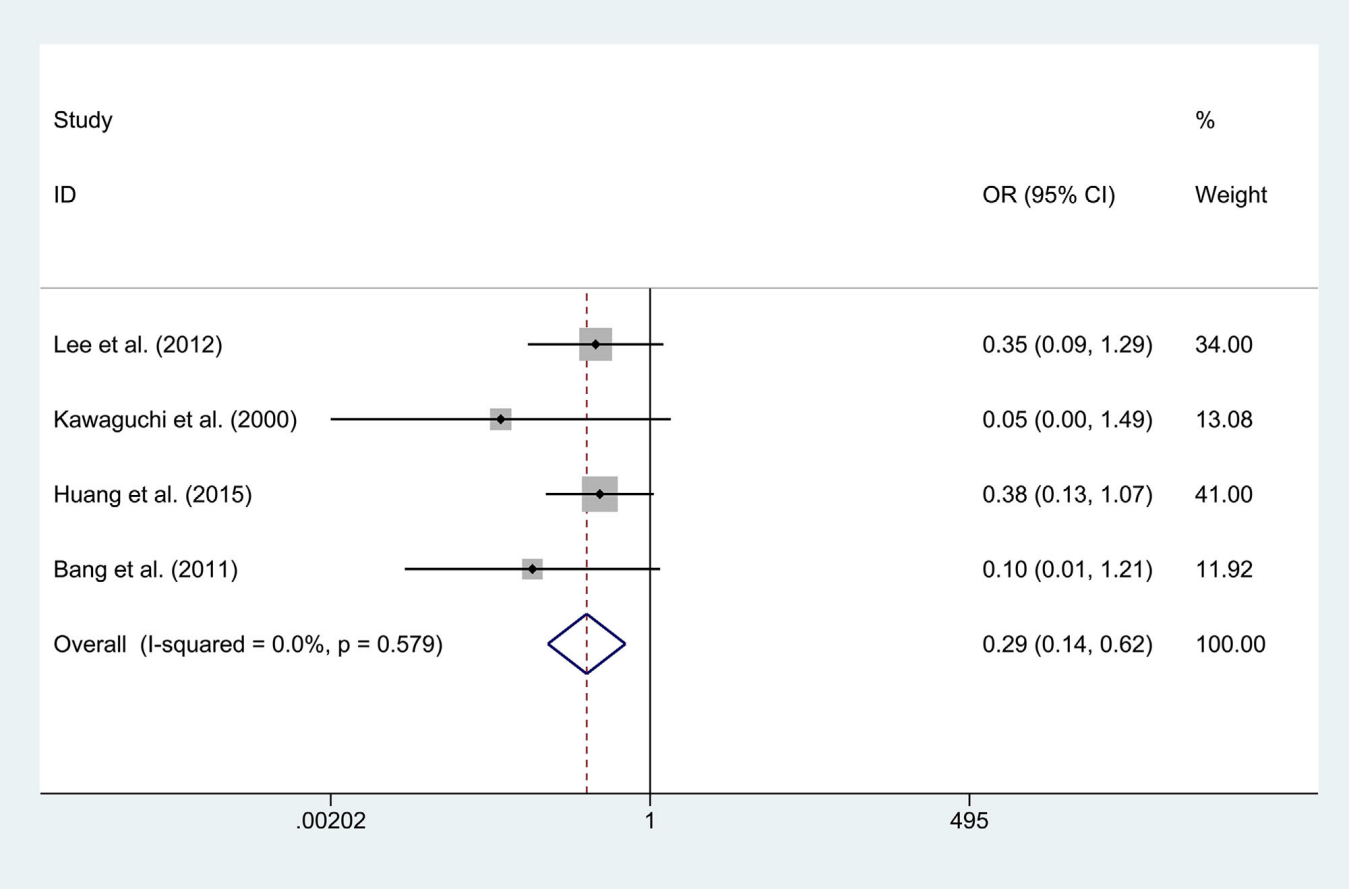
Forest plot for recurrence stroke between the indirect and direct bypass

**FIGURE 3:**
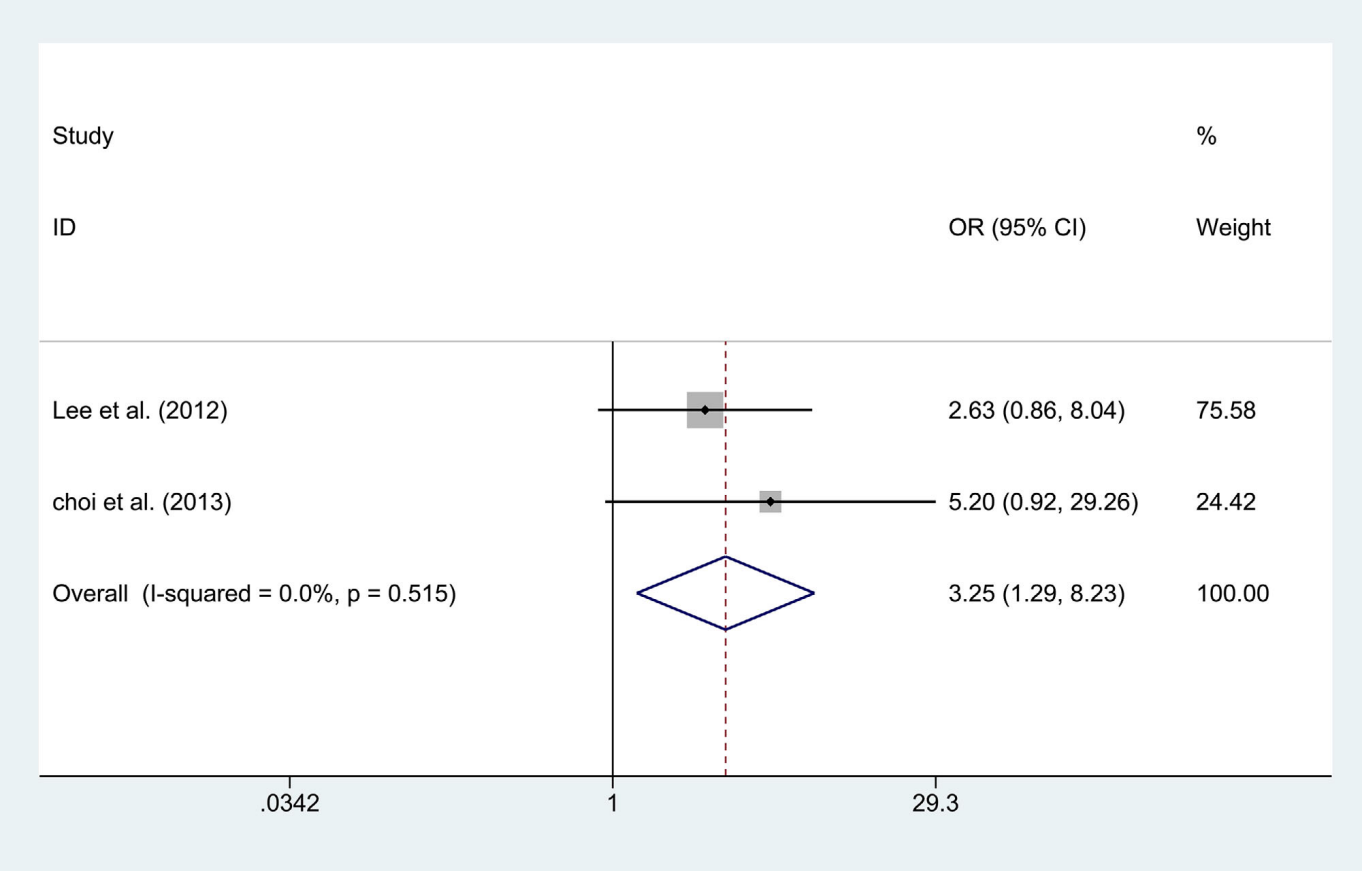
Forest plot for angiographic change between the indirect and direct bypass

Four publications (Bang et al., 2012; Huang et al., 2015; Kawaguchi et al., 2000; Lee et al., 2012) compare recurrent stroke between the DB and IB groups. We use a fixed‐effect model due to no obvious heterogeneity (*I* ^2^ = 0.0%). Besides, the patients in the IB group show a higher rate of recurrence stroke than the DB group (*p* = .001; Figure 2). There are two publications concentrating on the good angiographic change without obvious heterogeneity (*I* ^2^ = 0.0%) (Choi et al., 2013; Lee et al., 2012) and reveal a better angiographic change in the DB group (OR = 3.254, *p* = .013; Figure 3). There is no significant difference in the recurrent ischemic symptoms, recurrent hemorrhage, postoperative complication, and postoperative death between the two groups.

#### 4.4.2 CB versus IB

**FIGURE 4:**
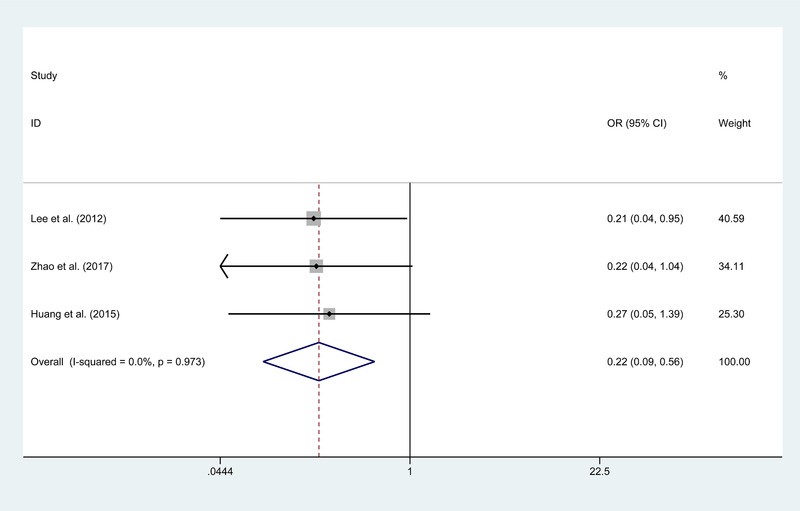
Forest plot for recurrence stroke between the combined and indirect bypass

Three works of the literature focus on recurrence stroke (Huang et al., 2015; Lee et al., 2012; Zhao et al., 2017) between CB and IB, and no obvious heterogeneity is observed. The patients after IB find a higher rate of recurrence stroke (*p* = .001; Figure 4). The postoperative complication is reported in six publications (Bang et al., 2012; Deng et al., 2018; Huang et al., 2015; Kim et al., 2012; Matsushima et al., 1992; Zhai et al., 2018) and shows no significant difference statistically (*p* = .483). Concerning the outcome of recurrent ischemic symptoms, postoperative death, and recurrent hemorrhage, the CB had no significant advantage over IB.

#### 4.4.3 CB versus DB

The recurrence strokes between the CB and DB group are compared in six studies (Bot et al., 2018; Golby et al., 1999; Huang et al., 2015; Lee et al., 2012; Matsushima et al., 1998; Zheng et al., 2019). The results indicated that there was no significant difference in the recurrence stroke (*p* = .232). Five studies mention the postoperative complications (Bang et al., 2012; Choi et al., 2013; Deng et al., 2018; Matsushima et al., 1998; Y. Zhao et al., 2018) and indicate that postoperative complications are irrelated to the surgery ways (*p* = .706). As for the recurrent ischemic symptoms, recurrent hemorrhage, postoperative death, and postoperative mRS (0‐2), no significant advantage is found between the two groups. More details about the outcome part are shown in Table 3.

**TABLE 3:** Results of the meta‐analysis

		Groups		Overall effect			Heterogeneity	
Outcome	Studies	DB OR CB	IB OR DB	Effect estimate	95% CI	*p*‐Value	*I* ^2^ (%)	*p*‐Value
Surgical treatment of moyamoya diseases: direct bypass (DB) surgery versus indirect bypass (IB) surgery								
Postoperative complications	4	19/90	14/69	OR, 1.075	0.451–2.564	.870	16.1%	.311
Postoperative death	5	9/152	14/69	RD, 0.010	−0.068 to 0.087	0.809	0.0%	.988
Recurrence stroke	4	12/152	33/121	OR, 0.292	0.137–0.619	**.001**	0.0%	.579
Recurrent ischemic symptoms	6	7/156	14/176	OR, 0.602	0.238–1.522	.283	0.0%	.881
Recurrent hemorrhage	6	7/159	18/119	RD, −0.126	−0.276 to 0.024	.100	75.7%	.001
Good angiographic change	2	13/39	13/95	OR, 3.254	1.287–8.226	**.013**	0.0%	.515
Surgical treatment of moyamoya diseases: combined direct and indirect bypass surgery versus indirect bypass surgery								
Postoperative complications	6	74/337	55/334	OR, 0.848	0.535–1.344	.483	0.0%	.520
Postoperative death	5	5/179	5/154	OR, 0.942	0.305–2.907	.917	0.0%	.614
Recurrence stroke	3	6/102	35/155	OR, 0.224	0.090–0.560	**.001**	0.0%	.973
Recurrent ischemic symptoms	6	13/197	19/301	RD, −0.029	−0.701 to 0.013	.174	0.0%	.998
Recurrent hemorrhage	7	8/348	20/421	RD, −0.700	−0.151 to 0.011	.089	83.8%	.0
Surgical treatment of moyamoya diseases: combined direct and indirect bypass surgery versus direct bypass surgery								
Postoperative complications	5	39/297	48/255	OR, 1.119	0.623–2.011	.706	0.0%	.739
Postoperative death	4	5/358	2/177	RD, 0.009	−0.018 to 0.035	.523	0.0%	.574
Recurrence stroke	6	16/164	6/135	RD, 0.040	−0.026 to 0.106	.232	0.0%	0.775
Recurrent ischemic symptoms	5	14/375	12/233	OR, 0.906	0.367–2.236	.831	0.0%	.790
Recurrent hemorrhage	7	10/393	8/252	RD, 0.003	−0.027 to 0.034	.831	0.0%	.866
Postoperative mRS (0‐2)	3	281/305	144/7	OR, 0.589	0.254 to 1.366	.217	43.1%	.172

Abbreviations: CB, combined direct and indirect bypass; CI, confidence interval; mRS, modified Rankin Scale; OR, odds ratio; RD, rate difference.

The bold value refers to *p* < 0.05.

## 5 DISCUSSION

MMD is a unique clinical entity, which is characterized by the progressive occlusion of the bilateral supraglenoid ICA. Despite MMD is firstly found in Japan, more and more patients with MMD have been found in China recently (Bao et al., 2015; X. J. Liu et al., 2015). To date, the most adopted revascularizations for MMD include DB, IB, and CB (Zhao et al. 2019), while the treatment prospects are limited since there is no known medical therapy that has been proven to be effective (Moussouttas & Rybinnik, 2020). DB seems to reduce the risk of stroke more than IB. Some studies supported using the CB strategy as the best alternative, employing both a direct STA‐MCA bypass and an IB such as EDAS or EDAMS (Amin‐Hanjani et al., 2013; Aoun et al., 2015). This study investigated the effects of various surgical treatments for MMD.

In the analysis, we compared the effects and safety of DB and IB for MMD and showed the DB group had a lower rate of recurrence stroke. Considering recurrent stroke prevention, DB has a huge advantage compared to IB. About angiographic change, we investigated that DB surgical treatment was better. Matsushima et al. did a retrospective study including 40 children who underwent either EDAS or STA‐MCA anastomosis with EMS. They reported that the DB resulted in better angiographic collateral filling and improved clinical outcomes (Matsushima et al., 1992). In another study conducted by Kawaguchi et al. comparing the outcomes between the two groups, the extent of revascularization was highest after DB than after only IB (Kawaguchi et al., 2000), and this study supported our angiographic finding.

Then, we compared the efficiency between CB and IB in patients with MMD. Despite the result indicated the patients in the IB surgery group did not have a higher postoperative complication rate, as for recurrence stroke, the IB group had a higher recurrence stroke rate. Previous studies indicated that IB can lead to surgical collaterals in about 40%−50% of adult patients, which may develop 3−4 months after surgery. CB has been found to have a better effect on revascularization compared with IB (Kuroda & Houkin, 2008). Kim et al. (2012) showed that CB was slightly superior to IB based on the extent of postoperative angiographic revascularization. Similarly, Noh et al. also revealed a favor of CB over IB (Noh et al., 2015). Those results supported the advantages of CB over IB, which is consistent with our research (Cho et al., 2014).

DB and CB surgery was reported to be more effective than IB in preventing rebleeding; however, the effect and superiority between DB and CB for MMD had barely been investigated yet (Y. Zhao et al., 2018). Therefore, this study was also concerned about the comparisons between CB and DB and revealed that there was no significant difference between CB and DB based on the aforementioned outcomes. It was noteworthy that these results were similar to those reported recently by Y. Zhao et al. (2018). Their results showed that CB did not bring additional risks during the postoperative period even though operation time was longer than DB.

This study had the following limitations: First, there were only seven factors used to analyze the effects and safety of different surgical revascularization of MMD may lead to unequal operation quality comparison. Second, some pieces of literature included in this meta‐analysis had a relatively small sample size. Finally, the focus of this meta‐analysis was to provide a short‐term outcome to clarify the value of DB, IB, and CB. Therefore, further attention should be paid to the long‐term efficacy of randomized controlled trials (RCTs) to determine the potential advantages of DB.

## 6 CONCLUSION

This meta‐analysis demonstrated a positive effect of using the direct and combined bypass to treat MMD compared to indirect bypass due to their lower rates of recurrence stroke.

## CONFLICT OF INTEREST

The authors declare no conflict of interest.

### PEER REVIEW

The peer review history for this article is available at https://publons.com/publon/10.1002/brb3.2356

## Data Availability

The datasets used and/or analyzed during this study are available from the corresponding author upon reasonable request.
